# A27 THE IMPACT OF MICROBIAL-DERIVED METABOLITES ON TYPE III INTERFERON SIGNALING IN INTESTINAL EPITHELIAL CELLS

**DOI:** 10.1093/jcag/gwae059.027

**Published:** 2025-02-10

**Authors:** T Olumade, C Lantin, Y Fedorova, R Nelson, O Ogungbola, O Fedorova, M Bording-Jorgensen, H Armstrong, D Santer

**Affiliations:** Department of Immunology, University of Manitoba, Winnipeg, MB, Canada; Department of Immunology, University of Manitoba, Winnipeg, MB, Canada; Department of Immunology and Department of Internal Medicine, University of Manitoba, Winnipeg, MB, Canada.Winnipeg, MB, Canada; Department of Immunology and Department of Internal Medicine, University of Manitoba, Winnipeg, MB, Canada.Winnipeg, MB, Canada; Department of Immunology, University of Manitoba, Winnipeg, MB, Canada; Department of Immunology and Department of Internal Medicine, University of Manitoba, Winnipeg, MB, Canada.Winnipeg, MB, Canada; University of Waterloo Department of Biology, Waterloo, ON, Canada; Department of Immunology and Department of Internal Medicine, University of Manitoba, Winnipeg, MB, Canada.Winnipeg, MB, Canada; Department of Immunology, University of Manitoba, Winnipeg, MB, Canada

## Abstract

**Background:**

Interferons (IFNs) are key cytokines that protect mucosal barriers. There are three types – types I, II and III. Unlike types I and II, which are pro-inflammatory, type III interferons (IFN-λs) are highly expressed in the gut and exert beneficial effects such as mucosal healing and dampening inflammation in mouse colitis models. Gut microbes can directly induce IFN-λ expression and prior studies have shown that microbial-derived metabolites can upregulate IFN-λ activity in the lungs. However, much less is known about the role of gut microbial-derived metabolites in the regulation of IFN-λ activity in the gut.

**Aims:**

We *hypothesized* that specific gut microbial-derived metabolites upregulate IFN-λ activity in human gut epithelial cells.

**Methods:**

Caco-2 cells were pre-treated for 2 hours with anaerobic whole microbe secretions (1-10% v/v) and microbial-derived metabolites, including short-chain fatty acids (SCFAs; acetate, butyrate, and propionate), a metabolic intermediate (succinate), and a tryptophan metabolite (kynurenine). Next, cells were treated with or without IFN-λ3 (20-50ng/ml) for 22 hours (n=3 biological repeats). IFN-stimulated genes (ISGs; *MX1* and *IFIT1*) were quantified by RT-qPCR. IFN-λR1 levels were quantified by flow cytometry.

**Results:**

Out of microbe secretions added from four gut microbes thus far, secretions from one pathobiont inhibited IFN-λ activity. All SCFAs tested had a concentration-dependent inhibitory effect on IFN-λ3 induction of ISGs (*MX1* and *IFIT1*; p<0.05). Succinate and kynurenine did not affect ISG induction by IFN-λ3 at any concentration tested. Only specific metabolites affected surface IFN-λR1 levels on Caco-2 cells.

**Conclusions:**

Our findings demonstrate that specific microbial-derived metabolites can regulate intestinal IFN-λ immune responses. Contrary to our hypothesis, some metabolites can downregulate IFN-λ activity. Knowledge from this study provides fundamental knowledge about IFN-λ regulation in the human gut with implications for how to promote optimal IFN-λ activity to promote gut health, such as in inflammatory bowel diseases.

**Funding Agencies:**

Children’s Hospital Research Institute of Manitoba, University of Manitoba

